# Chemical Characterization and Molecular Dynamics Simulations of Bufotenine by Surface-Enhanced Raman Scattering (SERS) and Density Functional Theory (DFT)

**DOI:** 10.1021/acs.jpclett.2c01300

**Published:** 2022-06-21

**Authors:** Xuanyi Wu, Maria Vega Cañamares, Ioanna Kakoulli, Santiago Sanchez-Cortes

**Affiliations:** † Department of Materials Science and Engineering, University of California, Los Angeles, Los Angeles, California 90095, United States; ‡ Molecular and Nano Archaeology Laboratory, University of California, Los Angeles, Los Angeles, California 90095, United States; § Instituto de Estructura de la Materia, IEM-CSIC, Serrano, 121, 28006 Madrid, Spain

## Abstract

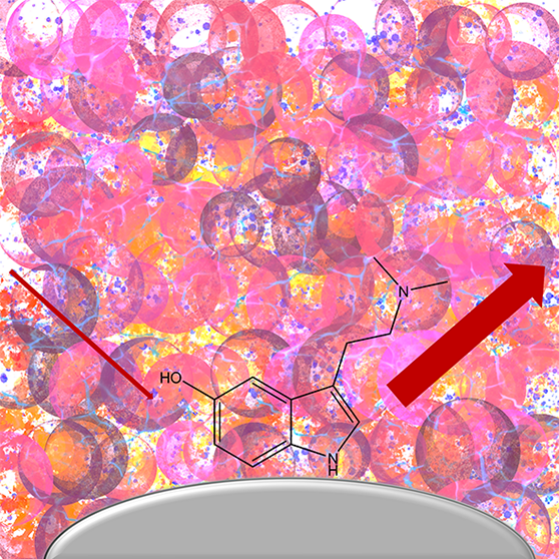

Bufotenine (5-hydroxy-*N*,*N*-dimethyltryptamine) is a natural tryptamine derivative with hallucinogenic activity. In this paper, we present novel chemical and molecular conformational analyses of bufotenine based on an experimental and theoretical approach integrating surface-enhanced Raman scattering (SERS) and density functional theory (DFT). For the first time, low concentrations of bufotenine in acetonitrile solutions were analyzed by SERS using two types of silver nanoparticle substrates synthesized via one- or two-step reduction processes. The vibrational characteristics of this molecule were verified by molecular dynamics simulations of Raman bands based on DFT. Here we demonstrate the potential of this integrated approach for the identification of bufotenine, a prominent hallucinogenic agent, establishing an innovative rapid and accurate sensing and characterization method of the identification of controlled substances at trace amounts.

Bufotenine (bufotenin, 5-hydroxy-*N*,*N*-dimethyltryptamine, or 5-HO-DMT), hereinafter BUF, a well-known hallucinogen,^1,2^ is a natural tryptamine derivative and dimethyl analogue to the significant neurotransmitter serotonin (5-hydroxytryptamine, or 5-HT) and similar to other types of tryptamine alkaloids such as psilocin (4-hydroxy-*N*,*N*-dimethyltryptamine, or 4-HO-DMT, associated with magic mushrooms), 5-methoxy-*N*,*N*-dimethyltryptamine (5-MeO-DMT), and *N*,*N*-dimethyltryptamine (*N*,*N*-DMT or DMT). In pre-Columbian populations, BUF-containing substances, such as algarroba (*Anadenanthera*) seeds which were the main ingredients in spiritual medicinal and snuffs, were found in drug paraphernalia in ancient burials.^2−6^ Other plant-origin sources containing BUF include the *Virola* tree, which contains 5-MeO-DMT as the psychoactive substance that produces BUF as the metabolite.^5,7^ BUF can also be found in fungi, such as mushrooms, and amphibians, such as toads.^1^

The main metabolic pathway of BUF is through oxidative deamination, with the aid of monoamine oxidase A (MAO-A), and into 5-hydroxyindoleacetic acid (5-HIAA).^8,9^ After injection, BUF tends to concentrate in animal lungs and hearts rather than in brains.^7^ Sudden deaths have been reported to be attributed to the consumptions of BUF-containing substances derived from toads,^10−12^ including the Western Indian aphrodisiac “Love Stone”^11^ and the traditional Chinese medicine “Chan Su”.^12^ BUF is a Schedule I drug in the United States,^13^ meaning that there is no accepted medical use of these chemicals and a high potential for abuse. In the past decade, BUF was also considered as a remedy for treating rabies.^14,15^ Therefore, the identification, detection, and studies of BUF are of scientific, forensic, criminological, social, and cultural importance. Here, we present a new analytical assay based on Raman and surface-enhanced Raman scattering (SERS) for the detection of BUF at trace amounts. To optimize the SERS spectra of BUF, a comprehensive study was performed by using different plasmonic silver (Ag) colloids and by varying the experimental conditions such as pH, excitation wavelength, and adsorbate concentration. Different SERS spectral profiles were obtained for BUF at different pH, thus indicating that the molecule can interact under different molecular structures with the Ag surface. Our experimental research was complemented and verified by theoretical calculations on conformation studies based on density functional theory (DFT).

The micro-Raman spectrum obtained at 532 nm excitation as well as the DFT calculated Raman spectrum is shown in Figure 1, and the main wavenumbers are displayed in Table 1. While weak peaks characteristic of BUF can be discerned in the experimental Raman spectrum, the spectrum is overwhelmed by background fluorescence. Two other spurious peaks shown at 570 and 1095 cm^–1^ correspond to the glass slide used as the sample holder.

**Figure 1:**
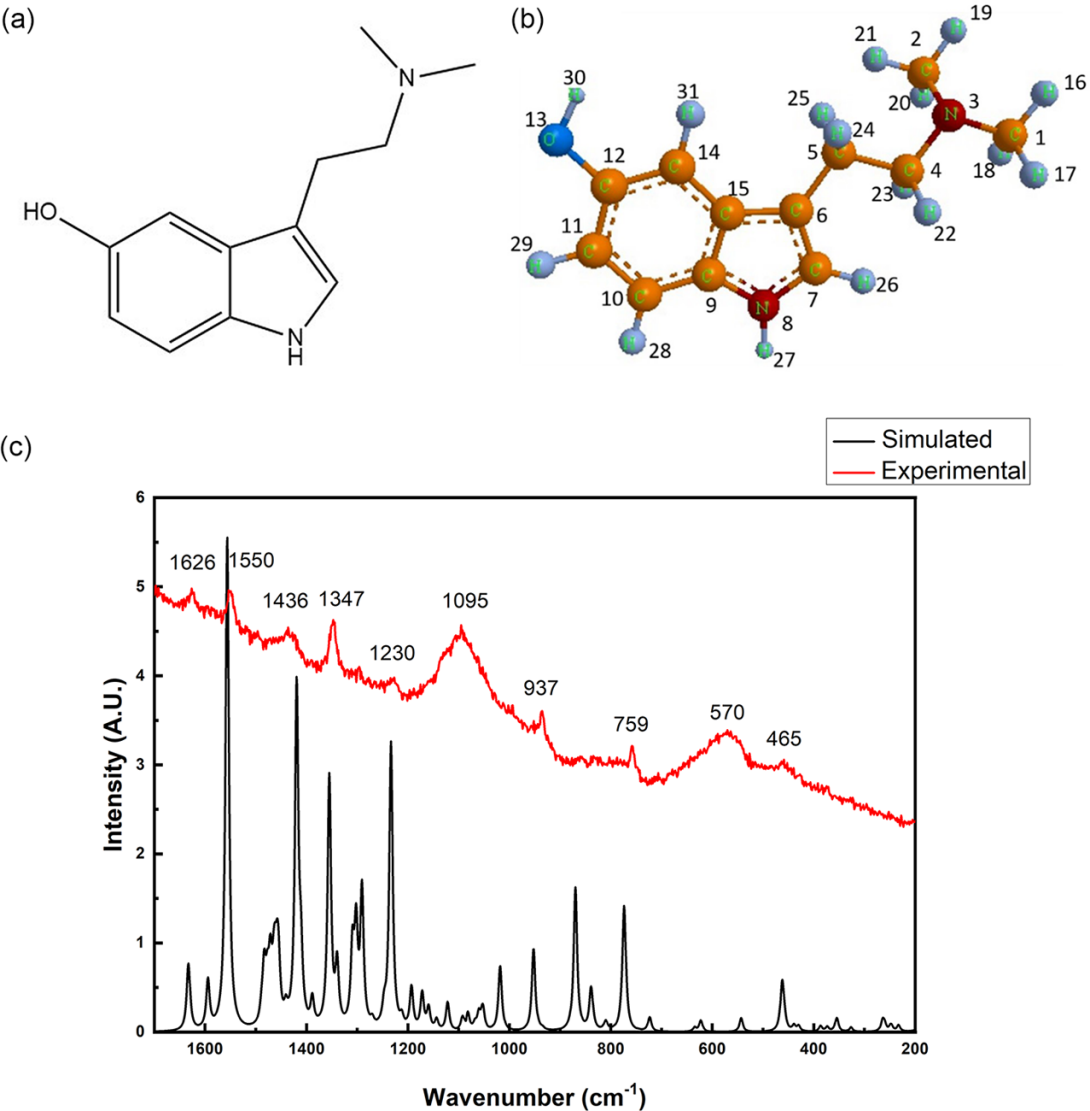
Molecular structure of bufotenine is depicted in (a) and (b). Experimental results based on the micro-Raman spectrum obtained with a 532 nm excitation laser (marked in red) and calculations based on DFT (marked in black).

**Table 1:** Band Assignment of Experimental (Raman and SERS) and Theoretical (DFT) Bands of BUF

Raman wavenumber (cm^–1^)					
micro-Raman^a^	SERS^a^ (type a)	SERS^a^ (type b)	SERS^a^ (type c)	DFT^a^ (scaled)	assignments^b^
1626 w			1628 sh	1633 w	ν(ring)
	1578 vs	1594 m	1565 s	1594 w	ν(ring)
1550 m				1556 vs	ν(C6=C7)/ν_ΙΙ_(ring)
	1488 vw	1488 vw	1485 vs	1483 sh	δ_as_(CH_3_)/δ_sc_(C4H_2_)
		1453 w		1462 m	ν(ring)
1436 w	1434 vs		1441 m	1441 vw	δ_s_(CH_3_)/δ_sc_(C4H_2_)
	1374 sh	1383 m		1389 vw	δ_w_(CH_2_)
1347 s	1358 m		1360 s	1355 s	ν(ring)/δ(OH)
	1319 vw		1319 m	1309 sh	δ_tw_(CH_2_)
1297 vw	1309 vw			1302 m	ν(ring)/δ(OH)
	1262 w			1271 vw	ν(C–N3)/ρ(CH_3_)
			1259 m	1247 sh	δ_tw_(CH_2_)/δ_II_(CH)
1230 w				1234 s	δ(CH)/δ_tw_(CH_2_)
			1211 w	1213 vw	δ(CH)/δ_tw_(C5H_2_)
	1170 w	1180 vw		1172 w	δ_tw_(CH_2_)/ν(C–N3)/ρ(CH_3_)
	1126 m	1130 m	1120 vs	1122 vw	δ(C10,11H)/δ_I_(CH)/δ(OH)
	1080 vw		1092 vw	1082 vw	ν(C4–C5)
			1074 w	1067 sh	ρ(CH_3_)
	1059 w			1060 vw	ν(C–N3)/ρ(CH_3_)
993 vw	1004 vw			1018 w	ν(C–N3)/δ(C–C5–C)/ρ(CH_3_)/δ(CH)
	973 vw		980 vw	953 m	δ(CH)/ν(C–O)
937 w	946 vw			935 vw	γ(C10,11H)
	846 w			839 w	γ(C14H)
			817 vw	810 vw	γ(CH)/ρ(CH_2_)
759 w				774 m	δ(ring)
			724 vw	724 vw	δ(ring)/ν(C–O)/δ(C–C5–C)
			661 vw	635 vw	γ(ring)
			620 w	623 vw	δ(ring)/δ(C–C5–C)
			583 w	543 vw	δ(ring)/δ(C–C5–C)
465 vw			465 m	462 w	δ(ring)/δ(C–N3–C)
	430 vw	432 vw		430 sh	γ_II_(ring)
	386 vw			387 vw	skeletal vibrations
	357 vw			355 vw	

a vw, very weak; w, weak; m, medium; s, strong; vs, very strong; sh, shoulder.

b ν, stretching; δ, in-plane bending; δ_tw_, twist deformation; δ_sc_, scissoring deformation; δ_w_, wag deformation; γ, out-of-plane bending; ρ, rocking; s, symmetric; as, asymmetric.

To overcome these challenges, improve the signal-to-noise ratio, and obtain additional information, SERS analyses of BUF on Ag-based plasmonic nanoparticles were performed. The application of the Ag colloids led to two important effects: (a) intensification of the Raman signal and (b) quenching of the fluorescence background. The response of BUF to SERS analysis was evaluated at different conditions: behavior of the molecule under pH 1–13 environment with two different excitation lines (532/633 nm) and by two types of silver substrates (silver nanostars, or AgNS, and silver nanospheres, or AgNSp). Experimental details are presented in the Supporting Information.

The main results are shown in Figure 2a (excitation at 532 nm on AgNS nanoparticles) and Figure 2c (excitation at 633 nm on AgNSp nanoparticles). Additional data are shown in Figure S2 (excitation at 633 nm on AgNS) and Figure S4 (excitation at 532 nm on AgNSp).

**Figure 2:**
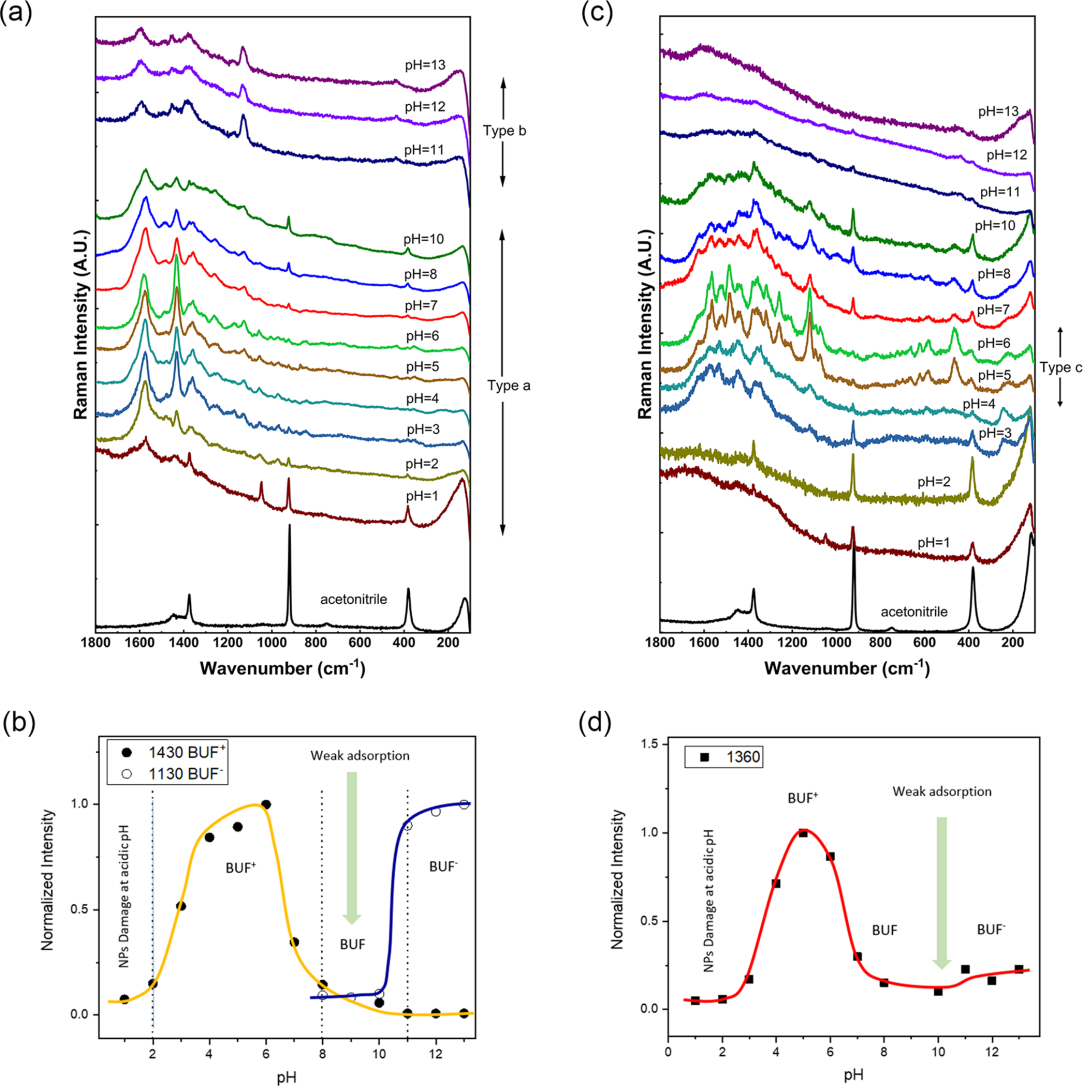
(a) SERS analysis at the pH range from 1 to 13, with a 532 nm laser and silver nanostars (AgNS). (b) Influence of pH in the intensity of SERS marker bands (at 1430 cm^–1^ for BUF^+^ in spectra type a and 1130 cm^–1^ for BUF^–^ in spectra type b) at 532 nm with AgNS. (c) SERS at pH range from 1 to 13 at a 633 nm laser on silver nanospheres (AgNSp). (d) Influence of pH in the intensity of SERS marker bands (at 1360 cm^–1^) at 633 nm with silver AgNSp.

The best results on signal intensity were obtained by using AgNS with an excitation of 532 nm (Figure 2a). Using this assay, we detected the vibrational modes of BUF at almost all pH values, although the absolute intensity is higher at pH between 4.0 and 6.0.

SERS spectra with two distinct types of spectral profiles were identified by varying the pH environment: spectra of type a, at pH lower than 8.0, and spectra of type b, at pH higher than 10.0 (Figure 2a). The existence of these two different spectra profiles is related to different interaction mechanism of BUF on the metal surface. Spectra of type a are dominated by two most intense bands appearing at 1575 and 1433 cm^–1^ showing an intensity increase when the pH decreases. The first band is characteristic of indole groups that are not interacting directly with the silver surface, whereas the second one is attributed to ν(ring) coupled with δ(CH_2_, CH_3_) vibrations, as deduced from the theoretical calculation (Table 1). The higher intensity of BUF at these pH intervals is due to the existence of a positive charge in the molecule, which is protonated in the amino side chain (−N^+^(CH_3_)_2_). Therefore, the protonated BUF (BUF^+^) can interact more strongly with the negatively charged surface, through the formation of electrostatic interactions between them. This is because of the existence of residual citrate ions (C_6_H_5_O_7_^3–^) on the Ag surface. In addition, this interaction can be strengthened by the establishment of H-bonds with the citrate ions on the surface; at acidic pH this is stronger due to the protonation of the carboxylic groups.

Spectra of type b exhibit bands at 1590, 1383, and 1130 cm^–1^. All these bands can be attributed to the indolic group but with shifted positions regarding the spectra observed at lower pH. This is due to the ionization of the −OH group at pH above 9.0–10.00, since the p*K* of phenolic groups falls in this pH interval. The affinity of the molecule is lower at high pH for two reasons: (a) the repulsive forces of the resulting indolate group and (b) the presence of OH^–^ ions that contribute to the passivation of the silver surface.

The SERS intensity undergoes a strong dependence on pH. Figure 2b shows the influence of the pH in the SERS signal intensity of BUF. The intensities of the BUF marker bands plotted in the figure were calculated from each spectrum upon background subtraction and subsequently normalized by using the acetonitrile band at 925 cm^–1^ whose relative intensity was set as 1. Four main regions can be distinguished, two of higher intensity, corresponding to the existence of the BUF^+^ (pH 3.0–8.0) and BUF^–^ (over pH 10.0) species, which exhibit a higher affinity for the surface. For the other two regions the SERS intensity collapsed because of a morphological damage of the Ag NPs at very low pH (below pH 2.0) and absence of charge in the molecule (neutral BUF species at pH 8.0–10.0). This neutral BUF species give rise to a slightly different spectrum a where the bands at 1575 and 1433 cm^–1^ are much weaker.

The study of the influence of pH on the SERS spectra of BUF indicates that the ionized forms display a higher affinity for the silver NP surface leading to a higher SERS enhancement (higher intensity). Conversely, the neutral form (BUF) exhibits a lower affinity giving rise to lower intensity spectra.

SERS spectra of BUF obtained at 633 nm by using AgNS nanoparticles are shown in Figure S2. At this excitation, SERS spectra show lower intensity compared to those registered at 532 nm, as deduced from the higher intensity of the acetonitrile bands. In addition, slightly different spectra are obtained at a pH where the cationic form BUF^+^ (pH 3.0–8.0) is expected. The observed differences between excitation lasers can be attributed to the coexistence of different species adsorbed on the surface and to the different resonance Raman conditions of these species when excited at different wavelengths. Furthermore, the anionic form is much weaker in the spectrum obtained at higher pH values (above 10.0).

Figure 2c displays the SERS spectra obtained for BUF when adsorbed on AgNSp nanoparticles at different pH and when exciting at 633 nm. Figure 2d displays the pH dependence of the marker bands at different pH. On these NPs, the behavior of BUF is different than that observed on AgNS, since only intense spectra of the molecule are obtained at a pH range where the cationic BUF^+^ is expected to exist on the silver surface (Figure 2d). This again is attributed to the higher affinity of the positively charged BUF^+^ in relation to the neutral form due to the net negative charge existing on the AgNSp surface. In general, the SERS spectra of BUF obtained on AgNSp are substantially different than those observed on AgNS for both the excitations at 532 and 633 nm. This is attributable to different chemical properties of these metal substrates and the different adsorption mechanism undergone by this molecule on surfaces capped with different molecules and ions on the surface. Therefore, in the specific case of the AgNSp substrates at 633 nm (Figure 2c), we have found that even the anionic form (BUF^–^) displays a low affinity, and this could be associated with the existence of chloride ions on the surface that are strongly attached to the AgNSp surface and that cannot be removed by BUF^–^.

On the other hand, spectra obtained in the region of the pH of highest intensity (5.0–6.0) display a different pattern regarding the spectrum a of Figure 2a, and we have referred to them here as spectra of type c. In Figure S3, spectra of types a and c are shown together for comparison. The type c spectra display intense bands at 465, 1120, 1259, 1360, 1485, and 1525 cm^–1^ which are very weak in the corresponding region of the AgNS nanoparticles. The presence of these bands indicates that the interaction of BUF with the silver takes place through the indole group because these bands are attributed to the indolic aromatic rings as also found in the case of tryptophane amino acid adsorbed on colloidal metals.^16−19^

A direct interaction of BUF through the N8–H with the surface is deduced from the appearance of the new band at 1525 cm^–1^ as also reported in previous works for similar chemical groups.^20^ In fact, the latter band is associated with stretching motions of the five-member ring coupled with NH bending. The intense bands at 1120 and 1259 cm^–1^, also attributed to the indolic moiety, corroborate this kind of interaction.

When the excitation laser at 532 nm is employed on AgNSp nanoparticles (Figure S4), a combination of the spectra a and c is observed at pH 5.0 and 6.0, where the cationic BUF^+^ species are expected to exist. This result points out again the coexistence of different BUF species adsorbed onto the surface of AgNSp. These species can be observed in the spectrum depending on the excitation laser. In fact, the molecule can interact by either the indole group or the alkylamino side chain as depicted in Scheme 1. A similar effect was observed in the case of other molecules such as alizarin.^21^

**Scheme 1:**
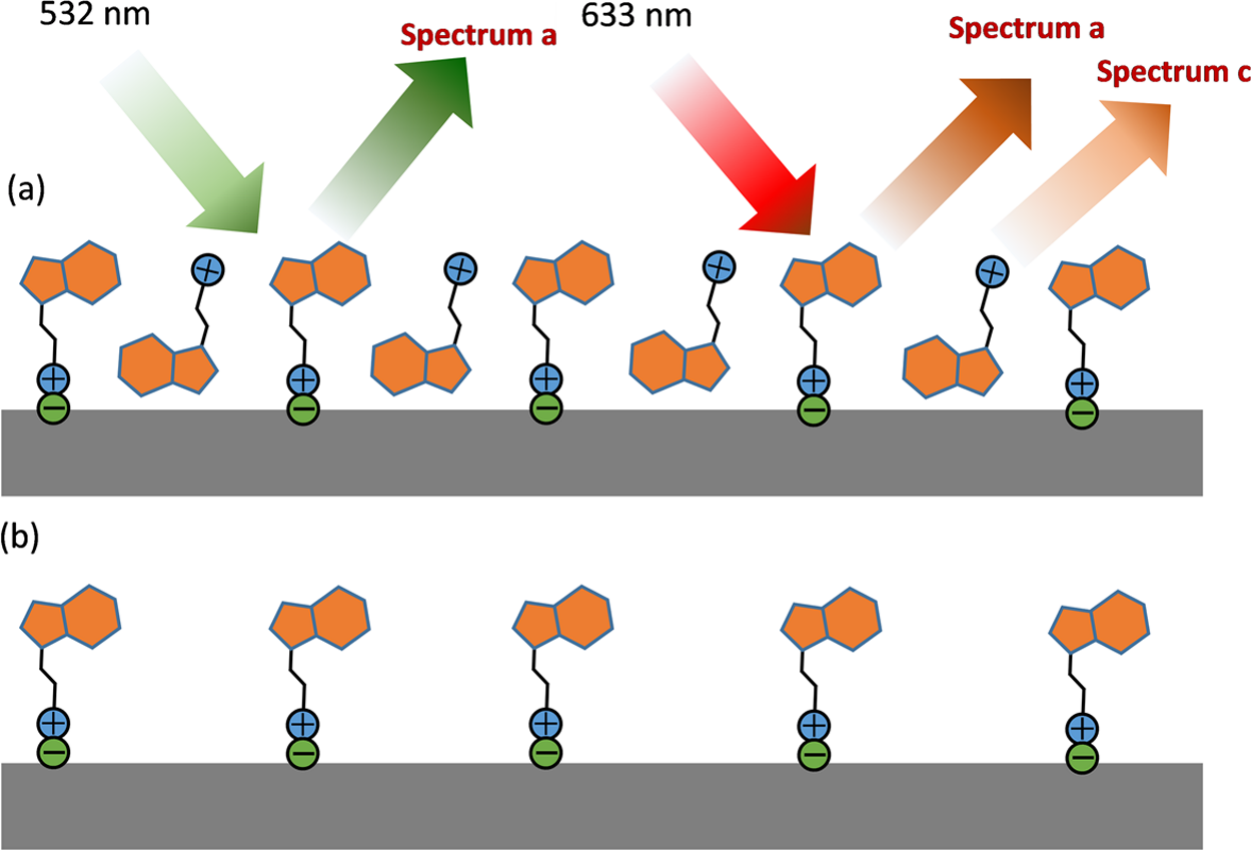
Schemes of the Adsorption of BUF^+^ at High (a) and Low Concentrations (b) and the Different Selectivity of Adsorbed Forms by Using Different Excitation Wavelengths at High Concentration, Which Accounts for the Spectral Variability

Another element of disparity in the SERS spectra of BUF is concentration. Because SERS is a detection technique based on the adsorption of a certain molecule on the interface of nanostructures, a different concentration in the medium is translated to a different surface coverage. On the other hand, the study of the SERS spectrum variation at different pH revealed different adsorption mechanisms of the drug on the metal surface. All these effects increase the complexity of the usual SERS intensity variation of the spectroscopic signal with the analyte concentration, as many different species are involved in the adsorption. To better analyze the effect of the drug concentration, we have focused this effect on the absolute intensity and on the spectral profile changes of the SERS spectra.

SERS spectra of BUF on AgNSp excited at 633 nm are shown in Figure 3 for concentrations ranging from 10^–4^ to 10^–8^ M. The analysis of the absolute intensity shows an increase from 10^–4^ to 10^–6^ M. However, on lowering further the drug concentration, the SERS intensity decreases. This behavior is produced by the effect of the adsorbate on the aggregation state of the NPs. At high concentrations the aggregation of NPs is very large, and the spectral signal can decline because of the lowering of the optical density in the sample. This effect induces a maximum of SERS intensity at 10^–6^ M concentration.

**Figure 3:**
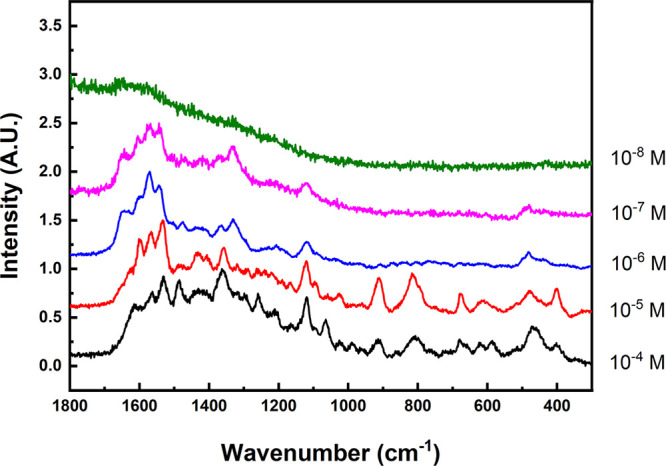
SERS spectra of BUF at different concentrations on AgNSp excited at 633 nm.

With regard to the spectral profile, a clear change in the relative intensity of bands is observed on decreasing the BUF concentration, specifically, a transition from spectra type c, seen at high concentration, to spectra similar to spectra type a, observed on lowering the concentration. In particular, the bands observed at 1082, 1165, and 1319 cm^–1^, attributed to ν(C4–C5), δ_tw_(CH_2_)/ν(C–N3)/ρ(CH_3_), and δ_tw_(CH_2_) according to our theoretical calculations, indicate that a predominant interaction through the alkylamino side chain takes place at low concentration. Under this interaction mechanism, the indole group is not interacting directly with the surface as corroborates the presence of bands at 1373, 1570, and 1595 cm^–1^ (Scheme 1b). However, at high concentrations, a different interaction mechanism appears to take place, involving a direct interaction of the indole group with the surface, as indicated by the presence of stronger bands at 1120, 1257, 1362, 1435, 1485, and 1530 cm^–1^ typical from the interacting indole with silver (Scheme 1a). Therefore, we have deduced that the predominant interaction of BUF with the silver NPs interface at low concentration is taking place through the ionic interaction of positively charged amino aliphatic side chain, which seems to be stronger that the direct interaction with the indole group. We have observed that a similar behavior regarding the higher affinity of alkyl amino in the tryptophane side chain to the surface of silver NPs.^20,22^

Using this protocol, we were able to demonstrate high sensitivity in the analysis for BUF with detection limit ∼10^–7^ M in acetonitrile with AgNSp nanoparticles and irradiation at the 532 or 633 nm laser (Figures 3 and S5). This corresponds to ca. 20 ppb in the case of BUF. The pH at 10^–4^ M measured 8.3 and, below this concentration, 6.5. The application of SERS in the detection of BUF provides a new reliable, high-sensitivity and high-specificity analytical assay for drug testing for both paleotoxicological studies and modern forensics. However, the possibility of finding different spectral profiles for the same drug depending on the chemical conditions or the excitation laser used in the analysis might complicate its identification in complex matrices.

Vibration modes deducted from Raman, SERS, and DFT are summarized in Table 1, and the optimized molecular geometry of BUF is displayed in Figure 4.

**Figure 4:**
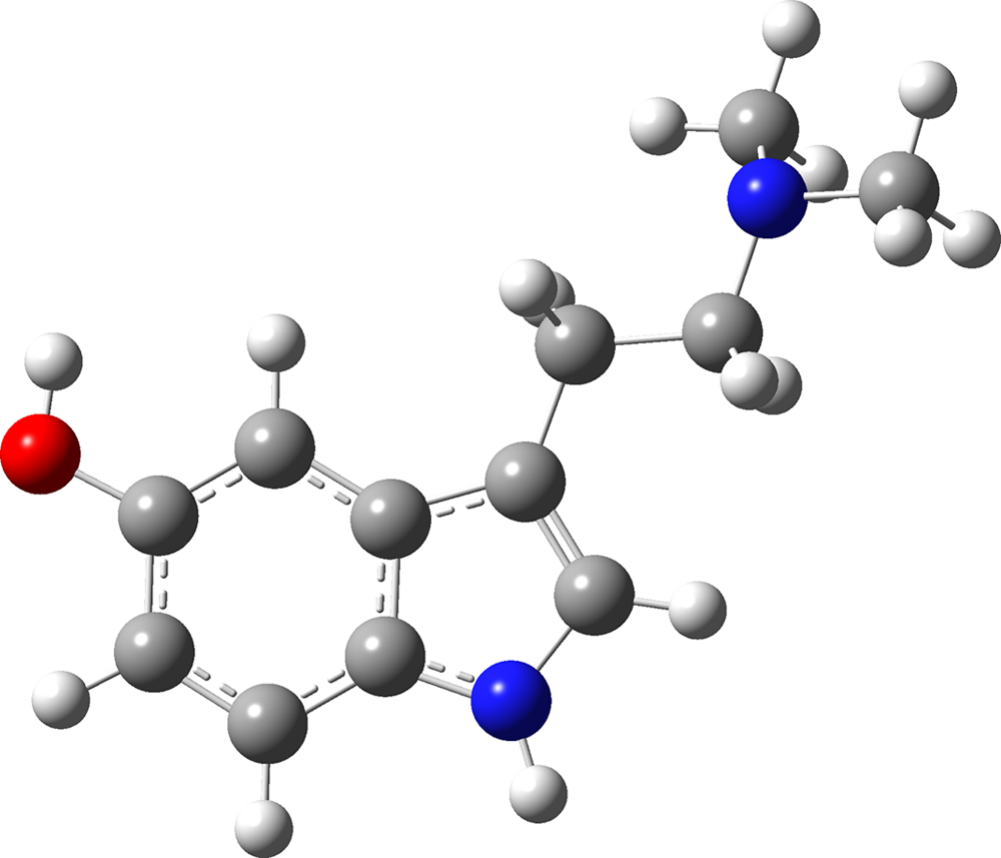
Optimized BUF geometry obtained by DFT calculations.

This research presents a comprehensive conformation analysis of the biomolecule BUF based on an experimental and theoretical approach integrating Raman, SERS, and DFT calculations, with special emphasis on its different structures adsorbed onto metal nanoparticles with different properties. Our protocol has demonstrated reliable and reproducible results for the detection and identification of BUF at trace amounts. Notable changes in SERS spectra of BUF depend on the environmental conditions, pH, excitation wavelength, the capping molecule existing in Ag colloids, and the drug concentration. The pH study allowed to obtain different spectra from BUF ionization species: cationic (BUF^+^), neutral (BUF), and anionic (BUF^–^) forms. SERS spectra afforded by each species substantially vary depending on the pH of the suspension. These important data should be considered when trying to identify this molecule in a complex molecular context. The analysis of the different SERS spectra served to obtain relevant structural information related to this drug, such as the p*K* of the different ionizations of BUF on the metal surface. The affinity for the Ag surface of the three different BUF species identified in the SERS spectra changes dramatically depending on the capping molecules or ions existing on the corresponding NPs. We have found that the affinity is much higher in the case of the cationic molecule (BUF^+^) because of the ionic interactions that these positive groups can establish with the negative charges provided by chloride or citrate ions existing on the surface of Ag NPs. BUF^+^ species are more abundant at pH 4.0–7.0. In this region of pH and on AgNSp, we have identified the presence of at least two different interaction mechanisms with the metal surface giving rise to different spectral patterns. One species is adsorbed through ionic interactions by the alkylamino side chain of BUF^+^, while the other is attached through the indole moiety. The ionic interaction is stronger than the indole direct linking, and it is the predominant one observed at low concentrations (under 10^–6^ M). The interaction through the indole group is only noted at concentrations over the last threshold. At these higher concentrations, the presence of several molecular forms implied in different interactions gives rise to different spectral patterns depending on the excitation wavelength. The assignment of the resulting adsorbed species was verified by the theoretical calculations conducted on this molecule.

## Experimental Methods

BUF was analyzed experimentally with SERS and theoretically with DFT. Two types of colloidal SERS substrates were used, including AgNS, based on procedures described by Garicia-Leis,^23,24^ and AgNSp, following the methods developed by Leopold and Lendl.^25^ DFT was performed by using the GAUSSIAN 09 package.^26^ Detailed materials information and experimental setup can be found in the Supporting Information.

## Supporting Information Available

Detailed materials, experimental and simulation setup; scanning electron microscopy (SEM) images and UV–vis extinction spectra of AgNS and AgNSp; SERS spectra of BUF at pH range from 1 to 13, at 633 nm excitation on AgNS; direct comparison between SERS spectra type a (on AgNS) and c (on AgNSp); SERS spectra of BUF at pH range from 1 to 13, at 532 nm excitation on AgNSp; SERS spectra of BUF at different concentrations on AgNSp excited at 532 nm (PDF)
Transparent Peer Review report available (PDF)
