# Supplementary material for: Chemical Characterization and Molecular Dynamics Simulations of Bufotenine by Surface-Enhanced Raman Scattering (SERS) and Density Functional Theory (DFT)
Source: J Phys Chem Lett. 2022 Jun 21;13(25):5831–7. doi: 10.1021/acs.jpclett.2c01300 (PMC9251765; doi:10.1021/acs.jpclett.2c01300)
Supplement: Supplementary file 1 — jz2c01300_si_001.pdf [file jz2c01300_si_001.pdf]

Supporting Information: Chemical Characterization and Molecular Dynamics Simulations of Bufotenine by Surface-Enhanced Raman Scattering (SERS) and Density Functional Theory (DFT)

*Xuanyi Wu[a,b], Maria Vega Cañamares[c], Ioanna Kakoulli[a,b], Santiago Sanchez-Cortes[c]**

[a]. Department of Materials Science and Engineering, University of California, Los Angeles (UCLA), CA 90095, USA.

[b]. Molecular and Nano Archaeology Laboratory, UCLA, CA 90095, USA.

[c]. Instituto de Estructura de la Materia, IEM-CSIC, Serrano, 121, 28006-Madrid, Spain.

*Email: s.sanchez.cortes@csic.es

BUF (4.9×10^{-4} M in acetonitrile) standard was purchased from Sigma Aldrich. Silver nitrate, hydroxylamine, hydroxylamine hydrochloride, sodium hydroxide, sodium citrate and nitric acid (all analytical grade) were purchased from Merck and Sigma-Aldrich.

Two types of silver nanoparticle (NP) were used for SERS enhancement: silver nanostars (AgNS) and silver nanospheres (AgNSp). The preparation for AgNS was based on the process

developed by Garcia-Leis[1,2]: A Erlenmeyer flask containing one magnetic stirrer was put on a stirring plate. 250 μL of Milli-Q water was added together with 250 μL NaOH solution (0.1M). 500 μL hydroxylamine with a molarity of $6.02x10^{-2}$ M was used as the first reducer. After 1 minute, the oxidizer $AgNO_3$ (9 mL, 10^{-3} M) was introduced, 1mL by 1mL. The second reducer, sodium citrate (100 μL, 1%wt) was added 5 minutes later. The solution was subsequently stirred for another 15 minutes. The preparation of AgNSp was based on the protocol described in Leopold-Lendl[3]. 90 mL Milli-Q water was poured in an Erlenmeyer flask with spinning. Subsequently, 0.0104 g $NH_2OH{\cdot}HCl$ was added to the flask, along with 300 μL NaOH solution at 1 M. The oxidizing agent, 10mL $AgNO_3$ (10^{-2} M) was then added drop by drop. The resulting solution was stirred for another 5 minutes before usage.

μ-Raman and SERS spectra were collected with two instruments: a Renishaw Raman In-Via spectrometer (Renishaw Iberica S.A.U., Gavá, Spain) equipped with a Leica microscope, and an electrically refrigerated CCD camera, using the 532 nm (Nd:YAG) excitation line, and a Renishaw RM-2000 spectrometer equipped with a 633 nm laser. The SERS spectra were the result of one scan registered with an integration time of 10 s and a spectral resolution of 2 cm^{-1}.

In μ-Raman analysis of the original solution, the resulting spectra were dominated by the vibrational signatures of the solvent, acetonitrile. 50 μL of the solution of BUF in acetonitrile were left to dry on a glass slide inside the fume hood. After the evaporation of the solvent, white powders of BUF were left on the glass surface.

Electron microscopy images were obtained with an Inspect S50 (FEI, Hillsboro, Oregon, OH, USA) scanning electron microscope (SEM). The analysis was performed with 20kV High Voltage.

Extinction spectra of the colloids were recorded with a Shimadzu 3600 spectrometer (Shimadzu Corp., Kyoto, Japan) equipped with a photomultiplier tube (PMT) for light detection in the UV-visible range and an InGaAs detector for the NIR. Samples were placed in quartz cells of 1cm optical path, after dilution to 30% volume ratio in Milli-Q water.

Experimental set-up: BUF and silver colloids were mixed in 1:3 ratios in small glass vials. NaOH and HNO_3 were used to vary the pH of the mixture. The pH was measured with pH strips. Spectra are normalized in order to have intensities in the range of 0 to 1. In the detection limit analyses, the pH was not further modified on top of the dilution process.

DFT calculations were performed in vacuum conditions using the B3LYP hybrid exchange correlation functional in combination with the 6-311++G** as a basis set. In two steps: in the first one, the optimization of the molecular geometry of BUF was carried out, while in the second one, the theoretical Raman spectrum was calculated. No imaginary wavenumbers were observed in the calculations. It is interesting to note that these calculations are carried out assuming that the molecule is isolated in the gas phase, while the Raman spectrum is collected from samples in their powder form. Thus, some differences in the relative intensities, as well as the frequencies of some of the lines are expected.

DFT calculations were carried out using the GAUSSIAN 09 package[4]. Scaling factors are commonly used in the calculated spectra to correct the correlation effects that are only partially accounted for in DFT[5]. In this work, 1 and 0.98 were used for wavenumbers below and above <1100 cm^{-1}, respectively.

All experimental and theoretical procedures were conducted at Instituto de Estructura de la Materia, Consejo Superior de Investigaciones Científicas (CSIC).

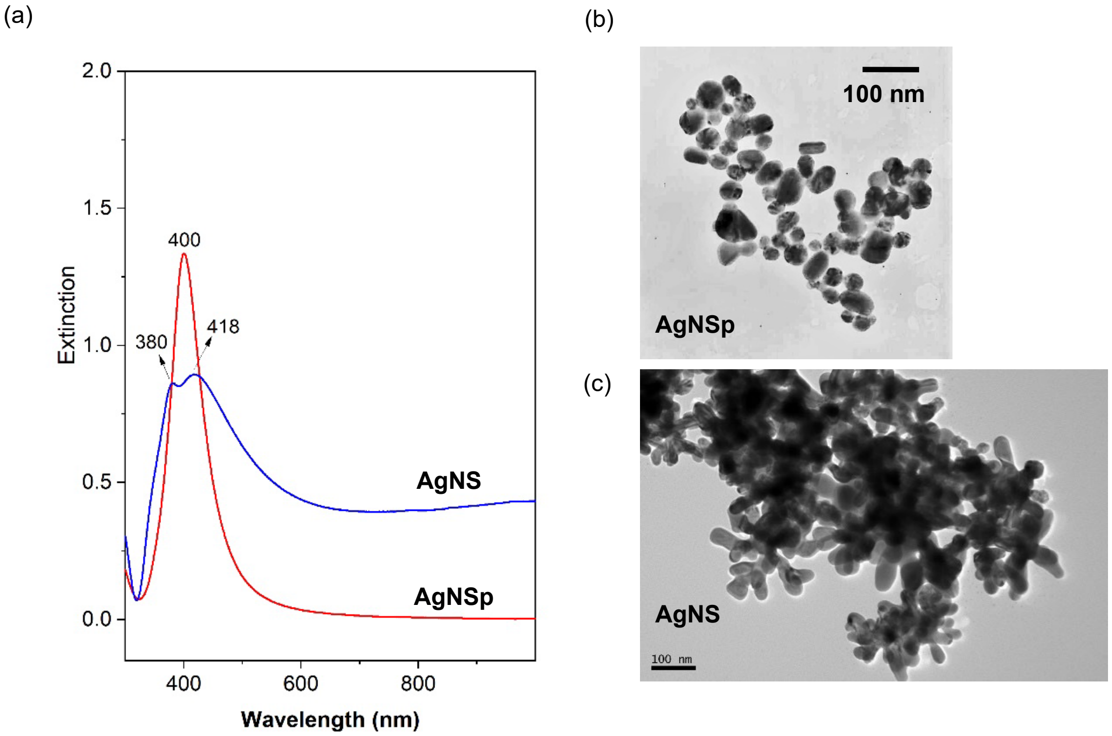

Figure S1. (a) Extinction spectra of AgNS and AgNSp nanoparticles; (b) SEM image of AgNSp NPs; (c) SEM image of AgNS NPs.

UV-visible spectra of the used NPs are shown in Fig. S1a. AgNSp shows a narrower extinction band peaking at 400 nm, while AgNS nanoparticles display two maxima at 380 and 418 nm, and a tail towards higher wavelengths. SEM images corresponding to these NPs are shown in Fig. S1b and c.

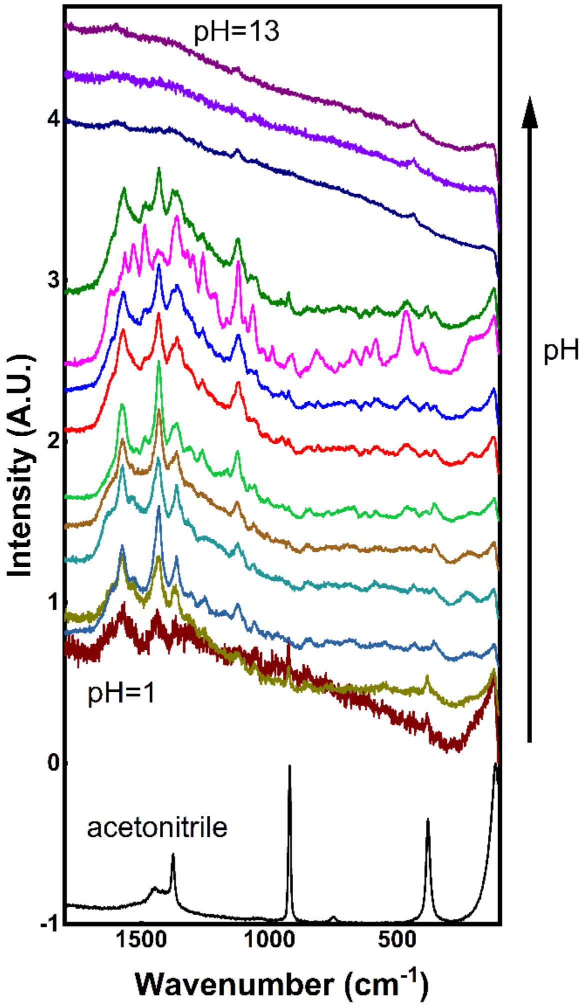

Figure S2. SERS spectra of BUF at pH range from 1 to 13, at 633 nm excitation on AgNS.

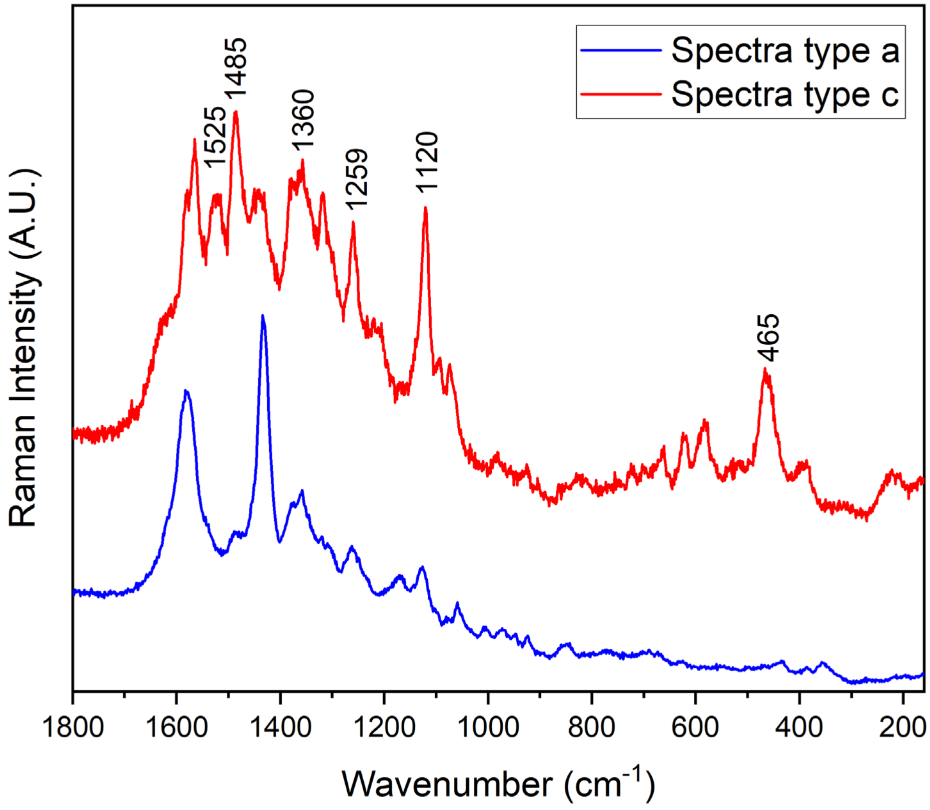

Figure S3. Comparison between SERS spectra type a (on AgNS) and c (on AgNSp).

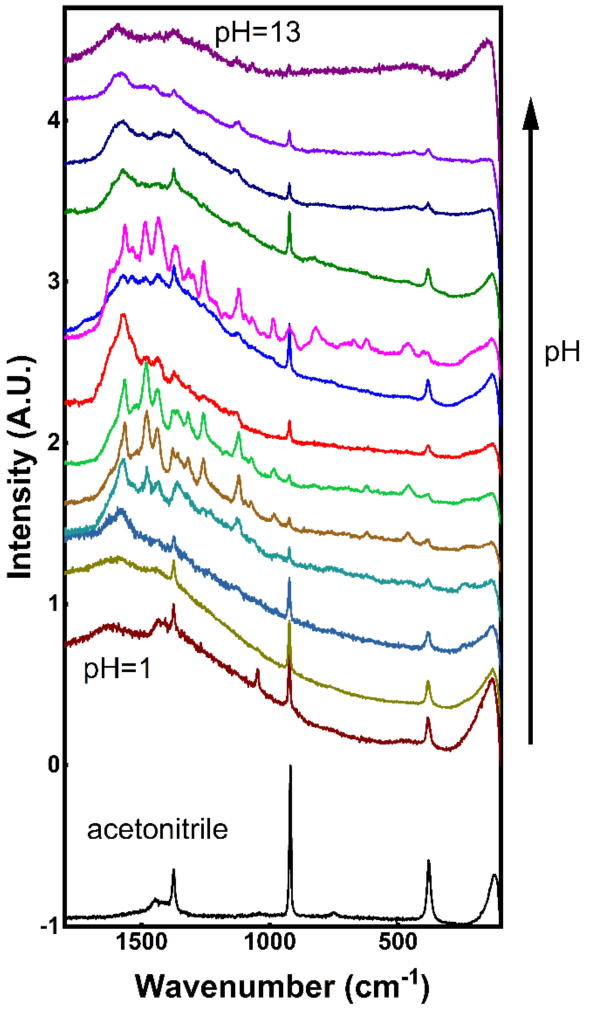

Figure S4. SERS spectra at pH range from 1 to 13, at 532 nm excitation line on AgNSp.

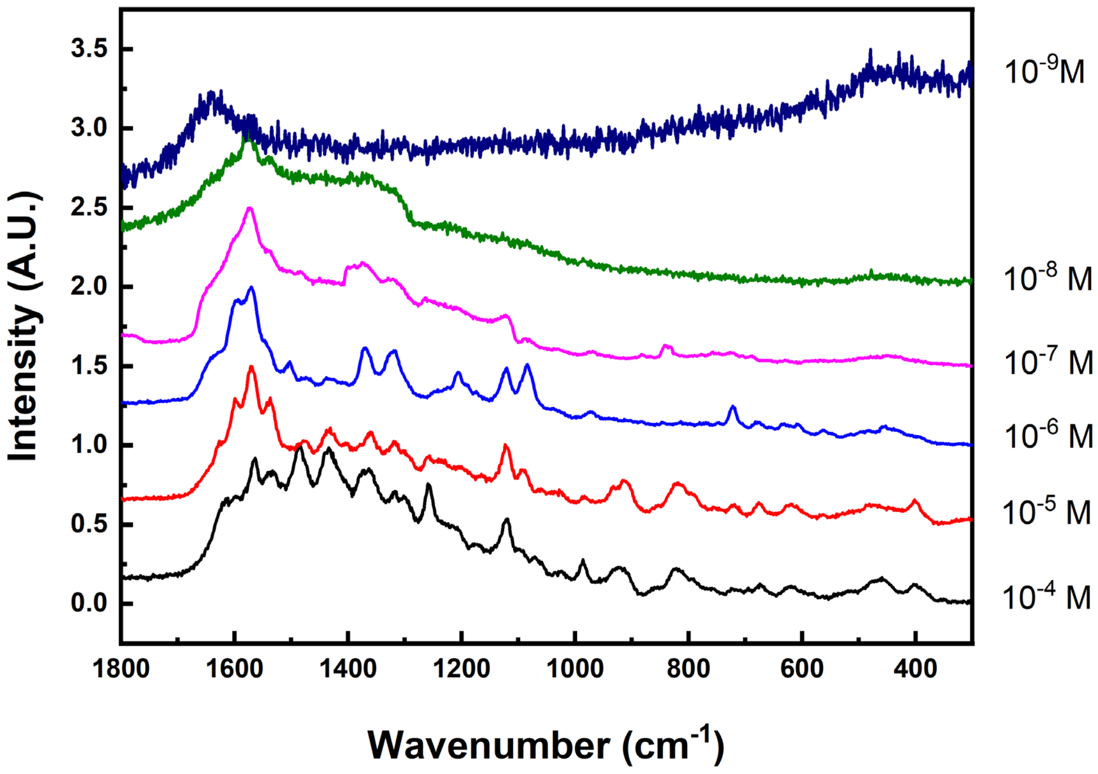

Figure S5. SERS spectra of BUF at different concentrations on AgNSp exciting at 532 nm.

REFERENCES

(1) Garcia-Leis, A.; Garcia-Ramos, J. V.; Sanchez-Cortes, S. Silver Nanostars with High SERS Performance. *J. Phys. Chem. C* **2013**, *117* (15), 7791–7795. https://doi.org/10.1021/jp401737y.

(2) Garcia-Leis, A.; Rivera-Arreba, I.; Sanchez-Cortes, S. Morphological Tuning of Plasmonic Silver Nanostars by Controlling the Nanoparticle Growth Mechanism: Application in the SERS Detection of the Amyloid Marker Congo Red. *Colloids Surf. Physicochem. Eng. Asp.* **2017**, *535*, 49–60.

(3) Leopold, N.; Lendl, B. A New Method for Fast Preparation of Highly Surface-Enhanced Raman Scattering (SERS) Active Silver Colloids at Room Temperature by Reduction of Silver Nitrate with Hydroxylamine Hydrochloride. *J. Phys. Chem. B* **2003**, *24* (107), 5723–5727. https://doi.org/10.1021/jp027460u.

(4) Frisch, M. J.; Trucks, G. W.; Schlegel, H. B.; Scuseria, G. E.; Robb, M. A.; Cheeseman, J. R.; Scalmani, G.; Barone, V.; Petersson, G. A.; Nakatsuji, H.; Li, X.; Caricato, M.; Marenich, A.; Bloino, J.; Janesko, B. G.; Gomperts, R.; Mennucci, B.; Hratchian, H. P.; Ortiz, J. V.; Izmaylov, A. F.; Sonnenberg, J. L.; Williams-Young, D.; Ding, F.; Lipparini, F.; Egidi, F.; Goings, J.; Peng, B.; Petrone, A.; Henderson, T.; Ranasinghe, D.; Zakrzewski, V. G.; Gao, J.; Rega, N.; Zheng, G.; Liang, W.; Hada, M.; Ehara, M.; Toyota, K.; Fukuda, R.; Hasegawa, J.; Ishida, M.; Nakajima, T.; Honda, Y.; Kitao, O.; Nakai, H.; Vreven, T.; Throssell, K.; Montgomery, Jr., J. A.; Peralta, J. E.; Ogliaro; Bearpark, M.; Heyd, J. J.; Brothers, E.; Kudin, K. N.; Staroverov, V. N.; Keith, T.; Kobayashi, R.; Normand, J.; Raghavachari, K.; Rendell, A.; Burant, J. C.; Iyerngar, S. S.; Tomasi, J.; Cossi, M.; Millam, J. M.; Klene, M.; Adamo, C.; Cammi, R.; Ochterski, J. W.; Martin, R. L.; Morokuma, K.; Farkas, O.; Foresman, J. B.; Fox, D. J. Gaussian 09 Revision A.02, 2016.

(5) Scott, A. P.; Radom, L. Harmonic Vibrational Frequencies: An Evaluation of Hartree-Fock, Møller- Plesset, Quadratic Configuration Interaction, Density Functional Theory, and Semiempirical Scale Factors. *J. Phys. Chem.* **1996**, *100* (41), 16502–16513.
