# Supplementary material for: Chemical Characterization and Molecular Dynamics Simulations of Bufotenine by Surface-Enhanced Raman Scattering (SERS) and Density Functional Theory (DFT)
Source: J Phys Chem Lett. 2022 Jun 21;13(25):5831–7. doi: 10.1021/acs.jpclett.2c01300 (PMC9251765; doi:10.1021/acs.jpclett.2c01300)
Supplement: Supplementary file 2 — jz2c01300_si_002.pdf [file jz2c01300_si_002.pdf]

jz-2022-01300w.R1

Name: Peer Review Information for "Chemical Characterization and Molecular Dynamics Simulations of Bufotenine by Surface-Enhanced Raman Scattering (SERS) and Density Functional Theory (DFT)"

First Round of Reviewer Comments

Reviewer: 1

Comments to the Author

In this article the authors undertake to investigate the surface-enhanced Raman spectra of the molecule Bufotenine (BUF) using Density Functional Theory (DFT) calculations as a guide for assignment of the Raman lines. Although BUF has no known therapeutic value, it does, however, act as a strong hallucinogen, and is therefore of considerable Forensic interest. Thus, a technique such as SERS can be of considerable value in trace analyses of residues left at crime scenes.

The SERS spectra are found to vary considerably with pH and therefore the authors undertake a detailed spectral approach examining the SERS spectra at varying pH conditions. They find three regions of the analysis, namely type a for pH< 8.0, type b for pH>10.0 and type c for pH range 5.0-6.0. Using the DFT results of type C they infer that the interaction of the Ag surface occurs through the indole group. Similar structural inferences are obtained though examination of type a and b regions. Such observations are of considerable value in Forensic applications since the spectral analysis is unambiguous and clearly demonstrated in this study. They are unlikely to be contested in a courtroom.

The article is clearly written and well documented. The DFT calculations lend considerable value to the results, and therefore the attention paid to these considerations a well-justified.

Due to the considerable value to the Forensic community, I highly recommend publication of this article.

Reviewer: 2

Comments to the Author

The manuscript presents results from the adsorption of bufotenine on silver nanoparticles at different pH conditions. The vibrational assignment was done based on DFT calculation. The text is well written and results are consistent, but some modifications have to be done before the publication.

1- The results presented in Fig. 3, where concentration was varying in SERS experiments, did not inform if the pH was monitored. If the answer is yes, what was such a value.

2- The Raman spectrum used in the first column in Table 1 was not presented neither in the main text nor in supporting information. Hence, it has to be included

3- The method for the normalization of Raman intensities for the construction of Fig. 2b and 2d through the use of acetonitrile band was not clear, since the preparation of SERS samples was not described in Experimental Section. It needs to be incorporated in the text.

4- The molecular structure of BUF depicted in Fig. 1a has to be modified in –N(CH3)2 region, where one methyl group was omitted.

5- Fig. 1b and all other Raman and SERS spectra presented in the manuscript have to be changed in accordance with IUPAC recommendation, by inverting the abscissa scales, starting from the greater wavenumbers to the lesser ones. In the same way, the use of “Wavenumber” in the legend of abscissa axes in Raman spectra is preferable to “Raman shift”

Author's Response to Peer Review Comments:

Xuanyi Wu
Department of Materials Science and Engineering, University of California, Los Angeles
410 Westwood Plaza
Los Angeles, CA, USA 90095
(424) 387-1381

June 3, 2022

Dear editors and reviewers,

Thank you very much for your kind feedback. We have modified the manuscript (Manuscript ID: jz-2022-01300w, Original Submission Date: 01-May-2022) accordingly and are wishing to submit for your further review. A copy of the manuscript and supporting information have also been uploaded with all changes marked in yellow. Below is a point-by-point response to the comments:

Editorial comments:

1) Author List: Please add a full header at top of the first page of the supplementary information file, which includes: title, full author list, and author affiliations (exactly as they appear in the main file,). Please format your author and affiliation information according to the guidelines in this link: https://pubsapp.acs.org/paragonplus/submission/author-address-information.pdf.

Thank you very much for the comment. The header has now been added to the supplementary information file (page S1).

2) Author Affiliations: Please include postal codes/country in the author affiliations in the publication file(s).

Thank you very much for the comment. The author affiliations have now been adjusted.

*3) References: In both the main file and the supporting information, fix the style of all references to use JPCL formatting (check all references carefully). ***JPC Letters reference formatting requires that journal references should contain: () around numbers, author names, article title (titles entirely in title case or entirely in lower case), abbreviated journal title (italicized), year (bolded), volume (italicized), and pages (first-last). Book references should contain author names, book title (in the same pattern), publisher, city, and year.*

Thank you very much for the comment. The reference style has now been adjusted and checked.

4) Supporting Information: Please number pages in the following format: "S1, S2…"

Thank you very much for the comment. The page numbers in SI have now been adjusted.

Reviewer #1 comments:

Thank you very much for the comments. We appreciate it.

Reviewer #2 comments:

1) The results presented in Fig. 3, where concentration was varying in SERS experiments, did not inform if the pH was monitored. If the answer is yes, what was such a value.

Thank you very much for the comment. The pH was not further modified on top of the dilution process in Figure 3. This pH at 10^{-4} M was measured 8.3, whereas at and below 10^{-5} M it was measured the same as the pH of the silver nanoparticle suspensions, which was 6.5. This information is now indicated in page 12, 2nd paragraph and in page S3, 2nd paragraph in SI.

2) The Raman spectrum used in the first column in Table 1 was not presented neither in the main text nor in supporting information. Hence, it has to be included.

Thank you very much for the comment. The data from the first column was presented in Figure 1c. We have now specified "µ-Raman" in the header of this column to avoid confusion in page 14. In addition, reference to the Table is indicated in page 4, 2nd paragraph.

3) The method for the normalization of Raman intensities for the construction of Fig. 2b and 2d through the use of acetonitrile band was not clear, since the preparation of SERS samples was not described in Experimental Section. It needs to be incorporated in the text.

Thank you very much for the comment. The SERS sample preparation was described in detail in the supporting materials in page S3, 2nd paragraph, and we have now added a sentence to point to that information before the discussion of the SERS results (page 4, 3rd paragraph). We have also modified the wording of the normalization process for clarification purposes (page 8, 2nd paragraph).

4) The molecular structure of BUF depicted in Fig. 1a has to be modified in –N(CH3)2 region, where one methyl group was omitted.

Thank you very much for the comment. This mistake was caused during the image formatting process and the image is now corrected.

5) Fig. 1b and all other Raman and SERS spectra presented in the manuscript have to be changed in accordance with IUPAC recommendation, by inverting the abscissa scales, starting from the greater wavenumbers to the lesser ones. In the same way, the use of "Wavenumber" in the legend of abscissa axes in Raman spectra is preferable to "Raman shift"

Thank you very much for the comment. All the spectra in the main text and in the supporting materials have now been modified accordingly.

Thank you very much for your consideration. We look forward to hearing from you.

Sincerely,

Xuanyi Wu
Ph.D. candidate,
Materials Science and Engineering Department
University of California, Los Angeles
